# An Artificial Pathway for *N*-Hydroxy-Pipecolic Acid Production From L-Lysine in *Escherichia coli*

**DOI:** 10.3389/fmicb.2022.842804

**Published:** 2022-03-08

**Authors:** Zhou Luo, Zhen Wang, Bangxu Wang, Yao Lu, Lixiu Yan, Zhiping Zhao, Ting Bai, Jiamin Zhang, Hanmei Li, Wei Wang, Jie Cheng

**Affiliations:** ^1^Meat Processing Key Laboratory of Sichuan Province, College of Food and Biological Engineering, Chengdu University, Chengdu, China; ^2^College of Science and Technology, Hebei Agricultural University, Cangzhou, China; ^3^College of Biological and Chemical Engineering, Guangxi University of Science and Technology, Liuzhou, China; ^4^Chongqing Academy of Metrology and Quality Inspection, Chongqing, China

**Keywords:** *N*-hydroxy-pipecolic acid, pipecolic acid, monooxygenase, artificial pathway, hydroxylate

## Abstract

*N*-hydroxy-pipecolic acid (NHP) is a hydroxylated product of pipecolic acid and an important systemic acquired resistance signal molecule. However, the biosynthesis of NHP does not have a natural metabolic pathway in microorganisms. Here, we designed and constructed a promising artificial pathway in *Escherichia coli* for the first time to produce NHP from biomass-derived lysine. This biosynthesis route expands the lysine catabolism pathway and employs six enzymes to sequentially convert lysine into NHP. This artificial route involves six functional enzyme coexpression: lysine α-oxidase from *Scomber japonicus* (RaiP), glucose dehydrogenase from *Bacillus subtilis* (GDH), Δ^1^-piperideine-2-carboxylase reductase from *Pseudomonas putida* (DpkA), lysine permease from *E. coli* (LysP), flavin-dependent monooxygenase (FMO1), and catalase from *E. coli* (KatE). Moreover, different FMO1s are used to evaluate the performance of the produce NHP. A titer of 111.06 mg/L of NHP was yielded in shake flasks with minimal medium containing 4 g/L of lysine. By this approach, NHP has so far been produced at final titers reaching 326.42 mg/L by 48 h in a 5-L bioreactor. To the best of our knowledge, this is the first NHP process using *E. coli* and the first process to directly synthesize NHP by microorganisms. This study lays the foundation for the development and utilization of renewable resources to produce NHP in microorganisms.

## Introduction

Plant metabolites play an important role in plant defense, since they can directly harm attacking pathogens, preventing pathogens from entering plant tissues (Chezem et al., 2017). Systemic acquired resistance (SAR) is one of the inducible forms of defense in plants, which produces long-lasting and broad-spectrum immunity against secondary infections at remote locations other than the initial infection site (Fu and Dong, 2013). In dicotyledonous and monocotyledonous plants, enhanced disease resistance is very effective in suppressing infections caused by biological and semibiological vegetative pathogens (Li et al., 2016). Many compounds have been identified as mobile signals of SAR, including salicylic acid (Metraux et al., 1990), methyl salicylate, pipecolic acid (Pip) (Ding et al., 2016), azelaic acid (Vicente et al., 2012), glycerol-3-phosphate (Chanda et al., 2011), nicotinamide adenine dinucleotide, *N*-hydroxy-pipecolic acid (NHP) (Guerra and Romeis, 2020), nicotinamide adenine dinucleotide phosphate, and dehydroabietinal (Chaturvedi et al., 2012).

Expanding the product range of renewable feedstock is critical to achieving a viable bio-based economy. With the great development of metabolic engineering and synthetic biology, more and more high-value chemicals can be produced from renewable raw materials by natural or artificial pathways in microorganisms (Cheng et al., 2021a). Recently, many important useful chemicals, such as ectoine (Zhang S. et al., 2021), itaconic acid (Elmore et al., 2021), naringenin (Zhang Q. et al., 2021), ε-caprolactone (Xiong et al., 2021), curcuminoids (Rodrigues et al., 2020), cadaverine (Ting et al., 2021), 5-hydroxyvaleric acid (Sohn et al., 2021), tyrian purple (Lee et al., 2021), 1,5-pentanediol (Cen et al., 2021), isobutanol (Yu et al., 2019), and pyruvic acid (Luo et al., 2020) have been obtained in microorganisms. There are many effective strategies that have been developed and used to improve the production of target chemicals, such as enzyme engineering (Ting et al., 2021), cofactor engineering (You et al., 2021), transcription factor engineering (Kang et al., 2021), promoter engineering (Gao et al., 2020), modularity engineering (Cen et al., 2021; Osire et al., 2021), ribosome binding site engineering (Wang et al., 2018), pathway engineering (Wang et al., 2021), fine-tuning gene expression (Wang et al., 2015), biosensor technology (Li et al., 2019), high-through screening (Zeng et al., 2020), and so on.

Recent research have shown that the lysine-derived metabolites Pip and SAR signal molecule NHP are key activators of the *Arabidopsis* system immunity, and their endogenous biosynthesis are essential for pathogen-induced SAR (Hartmann and Zeier, 2018). Pip is a cyclic amino acid, a common lysine metabolite in plants and animals (Pérez-García et al., 2019). Pip is an important precursor of some drugs, such as bupivacaine, rapamycin (Eggeling and Bott, 2015), and sandramycin (He, 2006). Importantly, Pip was a compatible solute for *Escherichia coli*, *Silicibacter pomeroyi*, and *Sinorhizobium meliloti* (Gouesbet et al., 1994; Gouffi et al., 2000).

Three synthetic pathways for producing Pip from L-lysine have been established (Han et al., 2020). The first synthetic route for Pip production was Δ^1^-piperidine-2-carboxylic acid (Pip2C)-mediated route (Tani et al., 2015). One-pot process of Pip production was established for expression of lysine α-oxidase (RaiP), Pip2C reductase (DpkA), and glucose dehydrogenase (GDH), which produced 45.1 g/L of Pip (Tani et al., 2015). Cheng et al. (2018) reported that the expression of lysine permease (LysP) can effectively increase the titer of Pip to 46.7 g/L. The second synthetic pathway was the Δ^1^-piperidine-6-carboxylic acid (P6C)-mediated route (Perez-Garcia et al., 2016). Pip (1.81 g/L) was accumulated in *Corynebacterium glutamicum* with overexpression of L-lysine 6-dehydrogenase (LysDH) and pyrroline 5-carboxylate reductase (ProC) (Perez-Garcia et al., 2016). Moreover, Perez-Garcia et al. (2017) found that *C. glutamicum* PIPE4 could utilize glycerol, starch to produce 14.4 g/L of Pip. The third synthetic pathway for Pip synthesis was the lysine cyclodeaminase (LCD)-mediated route (Ying et al., 2019). Han et al. (2020) expressed the LCD and optimized the expression vector and culture conditions, which produced 93.51 g/L of Pip.

The natural occurrence of SAR signal molecule NHP in organisms was first discovered in *Arabidopsis* (Hartmann and Zeier, 2018). NHP accumulated significantly after *Pseudomonas syringae* infection in locally inoculated *Arabidopsis* (Hartmann and Zeier, 2018). The biosynthesis of NHP in *Arabidopsis* involved AGD2-like defense response protein 1 (ALD1), SAR-deficient 4 (SARD4), and flavin-dependent monooxygenase 1 (FMO1) (Hartmann and Zeier, 2019). External application of NHP can improve the disease resistance of crops and achieve the purpose of disease prevention and control (Holmes et al., 2019). The phylogenetic analysis of the open genome based on the sequence information of *Arabidopsis* shows that homologs of the core genes of the NHP biosynthesis pathway are widespread throughout the plant kingdom (Holmes et al., 2019). Holmes et al. (2019) found that NHP accumulated in tobacco and tomato when infected with DC3000 bacteria. Therefore, monocots and dicots have the ability to trigger the biosynthesis of NHP in response to the infection of exogenous microorganism.

H_2_O_2_ could be exported from the cell, but its rapid production could lead to excessive accumulation of H_2_O_2_ in host cells, which would bring harmful effects (Korshunov and Imlay, 2010). Lv et al. (2018) reported that the activity of vanillyl alcohol oxidase PsVAO was significantly decreased over 20 μM of H_2_O_2_ by incubating PsVAO in H_2_O_2_. Hossain et al. (2016) established a whole-cell biotransformation system to form pyruvic acid in *E. coli* expressing L-amino acid deaminase. Finally, 14.57 g/L of pyruvic acid was achieved in the biocatalytic system (Hossain et al., 2016). Zhou et al. (2021) introduced a catalase ScCTA1 to scavenge H_2_O_2_ to further improve the titer of coniferyl alcohol to 53.90 g/L. Moreover, some catalases from *Saccharomyces cerevisiae* are introduced into *Hansenula polymorpha* and *Pichia pastoris* to scavenge harmful H_2_O_2_ (Gellissen et al., 1996; Payne et al., 1997).

**FIGURE 1:**
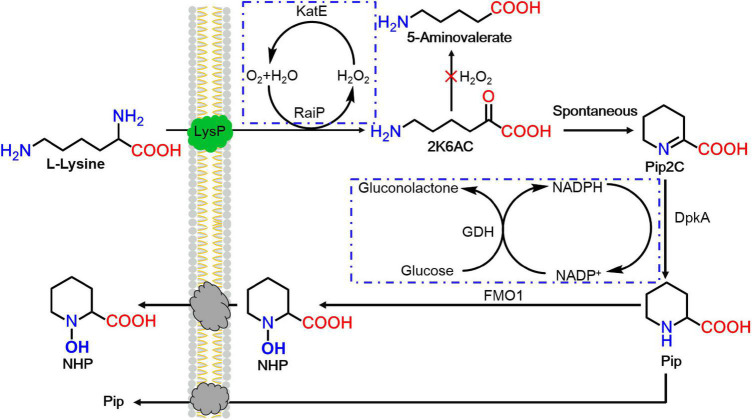
The heterogeneous pathway for *N*-hydroxy-pipecolic acid (NHP) production from lysine in *Escherichia coli*. 2K6AC, 2-keto-6-aminocaproate.

In this work, an artificial route for the production of SAR signal molecule NHP with RaiP, DpkA, GDH, LysP, FMO1, and catalase (KatE) overexpression was first successfully established in *E. coli*, as seen in Figure 1. First, lysine was oxidatively deammoniated, cyclized spontaneously, and reduced to generate Pip by RaiP and DpkA (Cheng et al., 2018). Subsequently, Pip was hydroxylated by FMO1 to form NHP. Engineering NHP biosynthesis into microorganism would be an attractive approach to enhance a plant’s exogenous ability to respond to pathogens.

## Materials and Methods

### Strains and Plasmids

Bacterial strains and plasmids used in this study are shown in Table 1. *E. coli* BL21(DE3) was used as the host strain to produce NHP. Strain BL21(DE3) with *cadA* gene knocked out was conducted in our previous studies (Cheng et al., 2018). The plasmid pTrc99a-*raip*-*dpkA*-*gdh*-*lysP* was constructed in our previous work (Cheng et al., 2018). The nucleotide sequences of lysine α-oxidase gene *raip* from *Scomber japonicus*, Pip2C reductase gene *dpkA* from *Pseudomonas putida*, glucose dehydrogenase gene *gdh* from *Bacillus subtilis*, and lysine permease gene *lysP* from *E. coli* are available in the GenBank with accession numbers MG423617, MG423618, MG425967, and WP_000253273.1, respectively. Codon-optimized flavin-dependent monooxygenase 1 gene *fmo1* from *Arabidopsis* (*Arfmo1*) (Accession No. NP_173359.3), *Brassica rapa* (*Brfmo1*) (Accession No. XP_009149401.1), *Glycine max* (*Gmfmo1*) (Accession No. XP_003541317.1), and *Zea mays* (*Zmfmo1*) (Accession No. XP_008660479.1) were chemically synthesized and inserted into pZA22 to form plasmid pZA22-*Arfmo1*, pZA22-*Brfmo1*, pZA22-*Gmfmo1*, and pZA22-*Zmfmo1* with *Acc*65I restriction site, respectively. The *katE* gene was amplified from *E. coli* MG1655 with *Sph*I and *Xba*I restriction site, ligated to the pZA22-*Afmo1* vector to generate recombinant pZA22-*Afmo1-katE*. The sequences of all vector constructs were verified by Sanger sequencing (Sangon Biotech Co., Ltd., Shanghai, China). The recombinant strains are listed in Table 1.

**TABLE 1:** List of strains and plasmids used in this study.

Strains or plasmids	Relevant genotype or description	Source
Strains		
BL21(DE3)	Wild type	Yan et al., 2013
ML03	BL21(DE3)Δ*cadA*	Cheng et al., 2018
ML07	ML03 harboring pTrc99a-*raip*-*dpkA*-*gdh*-*lysP*	Cheng et al., 2018
ML071	ML03 harboring pTrc99a-*raip*-*dpkA*-*gdh*-*lysP* and pZA22-*Arfmo1*	This study
ML072	ML03 harboring pTrc99a-*raip*-*dpkA*-*gdh*-*lysP* and pZA22-*Brfmo1*	This study
ML073	ML03 harboring pTrc99a-*raip*-*dpkA*-*gdh*-*lysP* and pZA22-*Gmfmo1*	This study
ML074	ML03 harboring pTrc99a-*raip*-*dpkA*-*gdh*-*lysP* and pZA22-*Zmfmo1*	This study
ML08	ML03 harboring pTrc99a-*raip*-*dpkA*-*gdh*-*lysP* and pZA22-*Brfmo1-katE*	This study
Plasmids		
pTrc99a-raip-dpkA-gdh-lysP	pTrc99a carries a L-lysine α-oxidase gene (raiP) from *S. japonicus*, a Δ1-piperideine-2-carboxylase reductase gene (dpkA) from *P. putida*, a glucose dehydrogenase (gdh) from *B. subtilis*, and a lysine permease gene (lysP) from *E. coli*, AmpR	Cheng et al., 2018
pZA22-*Arfmo1*	pZA22 carries a flavin-dependent monooxygenase 1 gene from *Arabidopsis* (*Arfmo1*), Kan*^R^*	This study
pZA22-*Brfmo1*	pZA22 carries a flavin-dependent monooxygenase 1 gene from *B. rapa* (*Brfmo1*), Kan*^R^*	This study
pZA22-*Gmfmo1*	pZA22 carries a flavin-dependent monooxygenase 1 gene from *G. max* (*Gmfmo1*), Kan*^R^*	This study
pZA22-*Zmfmo1*	pZA22 carries a flavin-dependent monooxygenase 1 gene from *Z. mays* (*Zmfmo1*), Kan*^R^*	This study
pZA22-*Brfmo1-katE*	pZA22 carries a flavin-dependent monooxygenase 1 gene from *B. rapa* (*Brfmo1*) and a catalase gene (*katE*) from *E. coli*, Kan*^R^*	This study

### Culture Medium and Conditions

*Escherichia coli* ML03 cells carrying the corresponding plasmids were cultured in 100-ml flasks containing 10 ml of Luria–Bertani (LB) medium (containing 10 g/L of tryptone, 5 g/L of yeast extract, and 10 g/L of NaCl) with appropriate antibiotics for 12 h at 37°C and 220 rpm. Then 20 μl of the culture was transferred into a 100-ml flask containing 20 ml of medium (composed of 15 g/L of glucose, 10 g/L of tryptone, 5 g/L of yeast extract, 0.5 g/L of K_2_PO_4_⋅3H_2_O, 3 g/L of KH_2_PO_4_, 2.5 g/L of (NH_4_)_2_SO_4_, 0.5 g/L of MgSO_4_⋅7H_2_O, 0.1 g/L of FeCl_3_, 2.1 g/L of citric acid⋅H_2_O, 40 mM α-ketoglutarate, and 100 μg/ml of Amp and/or 50 μg/ml of kanamycin) at 37°C and 220 rpm. After the OD_600_ reached 0.6, 0.5 mM IPTG was added, and culture was continued at 30°C for 48 h. L-lysine, Pip, and NHP were measured by HPLC.

### Biotransformation of *N*-Hydroxy-Pipecolic Acid in a 5-L Bioreactor

The biotransformation of engineering strains for producing NHP were conducted in a 5-L bioreactor. The formula of the medium was 40 g/L of glucose, 7.5 g/L of K_2_HPO_4_⋅3H_2_O, 1.6 g/L of (NH_4_)_2_SO_4_, 1.6 g/L of MgSO_4_⋅7H_2_O, 0.00756 g/L of FeSO_4_⋅7H_2_O, 2 g/L of citric acid, 0.02 g/L of Na_2_SO_4_, 0.0064 g/L of ZnSO_4_, 40 mM α-ketoglutarate, 0.0006 g/L of Cu_2_SO_4_⋅5H_2_O, 0.004 g/L of CoCl_2_⋅6H_2_O, 100 μg/ml of Amp, and 50 μg/ml of kanamycin. For the biotransformation in a 5-L bioreactor, an *E. coli* colony was seeded in 25 ml of LB medium in a 250-ml flask and incubated at 37°C and 220 rpm for 12 h. A 1-L flask containing 280 ml of LB medium was inoculated with the preculture and incubated at 37°C for 12 h with shaking at 220 rpm. The medium [2.8 L in a 5-L fermenter (Baoxing, Shanghai, China)] was then inoculated with an aliquot (10%) of the seed culture for biotransformation at 30°C. The pH of the medium was adjusted at 6.8 by NH_3_⋅H_2_O during the biotransformation process. The biotransformation was carried out at 30°C. After the OD_600_ reached 18, 0.5 mM IPTG and 40 g/L of lysine were added. The samples were taken at specific time intervals, and the residual lysine, Pip, and NHP were analyzed using HPLC.

### Analytical Methods

Lysine, Pip, and NHP were analyzed and quantitated by HPLC (Agilent Technologies 1200 series, Hewlett-Packard). The sample was derived with phenyl isothiocyanate (PITC) for the detection of lysine (Cheng et al., 2020). Pip and NHP contents were analyzed by HPLC with a Chirex^®^ 3126 (D)-penicillamine LC column (4.6 × 250 mm) as described by Cheng et al. (2018). Samples were centrifuged and filtered using a 0.22-μm membrane.

## Results and Discussion

### Construction of a Heterogeneous Pathway for *N*-Hydroxy-Pipecolic Acid Production in *Escherichia coli*

In this study, a functional heterogeneous route for NHP production from lysine was first established in *E. coli* by a multienzyme expression system. The biosynthesis route is employed in Figure 1. Strain ML07 for the production of NHP precursor Pip was constructed previously (Cheng et al., 2018). The designed heterogeneous route of NHP consists of two steps. The first step was to convert lysine into Pip in strain ML07 with coexpression of RaiP, DpkA, GDH, and LysP. The second step was to convert Pip into NHP mediated by FMO1. Although NHP can be produced in *Arabidopsis*, tomato (Holmes et al., 2019), and cucumber (Schnake et al., 2020), it has not been reported in microorganisms. Hence, we tried to introduce a plant FMO1 from *Arabidopsis* (ArFMO1) in combination with Raip, DpkA, GDH, and LysP for NHP production from lysine. First, a plasmid pZA22-Arfmo1 was constructed. Then plasmids pTrc99a-raiP-dpkA-gdh-lysP and pZA22-Arfmo1 were introduced into *E. coli* ML03 to obtain the engineered strain ML071. As shown in Table 2, we investigated the production of NHP from lysine in strain ML071. When 2 g/L of L-lysine was fed to the recombinant *E. coli* ML071, 29.36 mg/L of NHP was produced after 12 h (Table 2). A titer of 86.48 mg/L of NHP could be obtained with 4 g/L of lysine addition after 48 h. Our data clearly proved that the heterogeneous pathway of NHP in *E. coli* is feasible.

**TABLE 2:** Production of *N*-hydroxy-pipecolic acid (NHP) from lysine by recombinant *Escherichia coli* ML071.

Strains	Time (h)	2 g/L of lysine		4 g/L of lysine	
			
		NHP production (mg/L)	NHP yield (g/g)	NHP production (mg/L)	NHP yield (g/g)
ML07	12	0	–	0	–
	24	0	–	0	–
ML071	12	29.36 ± 1.84	0.01 ± 0	53.57 ± 2.92	0.01 ± 0
	24	47.52 ± 2.66	0.02 ± 0	86.48 ± 3.28	0.02 ± 0

### Comparison of *N*-Hydroxy-Pipecolic Acid Synthesis by Different Flavin-Dependent Monooxygenases in *Escherichia coli* Strains

Although the function of AfFMO1 has been primarily verified in this study, the synthesis efficiency of NHP was far from satisfactory. It was presumed that other alternative enzymes from other sources might work better. FMO1s from different sources can catalyze the direct formation of NHP from Pip. To evaluate the performance of the four FMO1s in *E. coli*, four recombinant strains overexpressing different FMO1s, i.e., ArFMO1 from *Arabidopsis*, BrFMO1 from *B. rapa*, GmFMO1 from *G. max*, and ZmFMO1 from *Z. mays*, were constructed in this work.

**FIGURE 2:**
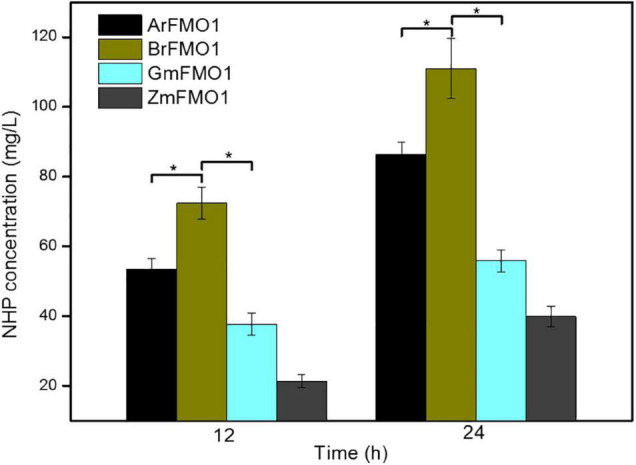
Comparison of NHP synthesis by different FMO1s in *E. coli* strains. NHP production at 12 and 24 h by strains ML071, ML072, ML073, and ML074, supplemented with 4 g/L of lysine as substrate. Statistics were performed by two-tailed Student’s *t*-test. *p < 0.05. Each experiment was done at least in triplicate. Error bars indicate standard errors of the means.

As shown in Figure 2, the strains ML073 (GmFMO1) and ML074 (ZmFMO1) resulted in even less NHP production than ML071 (ArFMO1) (as 55.78 and 39.88 mg/L, respectively). The results improved greatly in ML072 (BrFMO1), with an enhancement to 111.06 mg/L of NHP, which was nearly 28% higher than ML071 (ArFMO1). The results indicated that BrFMO1 is slightly more active than AfFMO1, GmFMO1, and ZmFMO1 toward Pip (Figure 2). As a control, *E. coli* ML03 carrying the pTrc99a-raiP-dpkA-gdh-lysP vector did not produce any NHP.

### One-Pot Biotransformation of *N*-Hydroxy-Pipecolic Acid Production at the 5-L Scale

**FIGURE 3:**
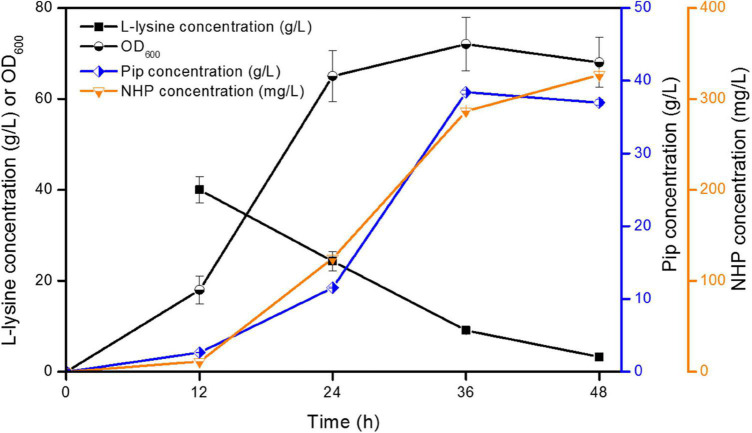
Time profiles of NHP production were investigated in *E. coli* ML08 in a 5-L fermenter. The experiments were conducted at 40 g/L of L-lysine. Each experiment was done at least in triplicate. Error bars indicate standard errors of the means.

However, strain ML072 also produces H_2_O_2_ by RaiP, which impairs cells and can further oxidize 2-keto-6-aminocaproate (2K6AC) to 5-aminovalerate (5AVA) as a byproduct (Cheng et al., 2021b). Therefore, we introduced *katE* encoding a catalase to decompose harmful H_2_O_2_ and eliminate its side effects in strain ML08. The biotransformation of NHP in *E. coli* ML08 was executed in this study, and the results are shown in Figure 3. The data showed that the biomass increased from OD_600_ 5.38 to 65 between 8 and 24 h. Pip (38.42 g/L) was produced, and 286.42 mg/L of NHP was accumulated after 36 h. Further increasing the fermentation time to 48 h could obtain a little higher NHP titer of 326.42 mg/L. At the same time, 36.98 g/L of Pip remained as a byproduct, indicating that the catalytic efficiency of BrFMO1 is not high and needs to be further improved. Under optimal biotransformation conditions, 326.42 mg/L of NHP was obtained in a 5-L bioreactor from 40 g/L of L-lysine in 48 h.

Although H_2_O_2_ is distributed in almost all organisms, when its concentration exceeds a certain threshold, it could cause adverse effects, such as cytotoxicity and enzyme inhibition (Valderrama, 2010). A high concentration of H_2_O_2_ would affect cell growth and limited the production of target products (Niu et al., 2014; Cheng et al., 2021b). Liu et al. (2017) found that catalase KatE was introduced to decompose H_2_O_2_, which increased the titer of α-ketoglutaric acid to 77.4 g/L. The concentration of 5-aminovalerate was further improved by expressing catalase KatE in a novel synthetic pathway in our previous study (Cheng et al., 2021b). Therefore, the elimination of H_2_O_2_ *in situ* is conducive to the production of the target product.

## Conclusion

In conclusion, we described a biotransformation system utilizing RaiP, DpkA, GDH, LysP, KatE, and BrFMO1 to convert L-lysine to SAR signal molecule NHP in *E. coli*. This artificial pathway in this work announces a greener and healthier production of SAR signal molecule NHP. An engineered *E. coli* strain with RaiP, DpkA, GDH, LysP, KatE, and BrFMO1 overexpression can produce 326.42 mg/L of NHP from L-lysine in a 5-L bioreactor. This microbial transformation system utilizes a renewable substrate and has simple culture conditions. As shown in Figure 3, Pip is produced as the main byproduct, which is not an efficient way to produce NHP. So the next goal is to improve the catalytic activity of BrFMO1, then to achieve a more efficient NHP production.

## Data Availability Statement

The original contributions presented in the study are included in the article/supplementary material, further inquiries can be directed to the corresponding authors.

## Author Contributions

ZL and ZW performed the experiments, analyzed the data, and drafted the manuscript. BW, LY, TB, JZ, and HL analyzed the data. ZZ, WW, and JC coordinated the study and finalized the manuscript. All authors contributed to the article and approved the submitted version.

## Conflict of Interest

The authors declare that the research was conducted in the absence of any commercial or financial relationships that could be construed as a potential conflict of interest.

## Publisher’s Note

All claims expressed in this article are solely those of the authors and do not necessarily represent those of their affiliated organizations, or those of the publisher, the editors and the reviewers. Any product that may be evaluated in this article, or claim that may be made by its manufacturer, is not guaranteed or endorsed by the publisher.
